# Control of stress and damage in structures by piezoelectric actuation: 1D theory and monofrequent experimental validation

**DOI:** 10.1002/stc.2338

**Published:** 2019-02-28

**Authors:** Juergen Schoeftner, Andreas Brandl, Hans Irschik

**Affiliations:** ^1^ Institute of Technical Mechanics Johannes Kepler University Linz Linz Austria

**Keywords:** control of fatigue and damage, dynamics of structures, piezoelectric actuation, stress control, structural control

## Abstract

This contribution presents novel results on feed‐forward control of stress in piezoelectric structures by means of piezoelectric actuation. For that sake, we focus on a one‐dimensional benchmark problem, a piezoelectric transducer that is excited by a piezoelectric stack actuator. We investigate the following problem: Is it possible to actuate the piezoelectric transducer in such a manner that the dominant axial stress component is nullified. In order to find a theoretical solution for this question, we discretize our system as a two‐degree‐of‐freedom (2DOF) model. The equations of motion are transformed into the differential equations for the inner forces by taking advantage of the constitutive relations, which relate displacement, stress, and electric field. Finally, we find a mathematical relation for the piezoelectric transducer excitation in order to annihilate the transducer force. A static and a frequency‐dependent approximate solution for the transducer actuation signal are derived. The latter solution reduces the inner force drastically in a certain frequency range. After numerical results for the force‐control algorithm are presented, we finally experimentally verify our theory: First, the force‐controlled configuration is exposed to a monofrequent harmonic excitation test run for 30 min, showing no sign of fatigue or material failure, because the transducer force is below the ultimate tensile strength. Then, the system is excited by the same harmonic excitation again, but the control signal for the piezoelectric transducer is turned off. The result is a visible damage of the piezoelectric transducer, leading to a significant change of the first eigenfrequency.

## 1 INTRODUCTION

Smart or intelligent structures are equipped with multifunctional materials. These structures often take advantage of the piezoelectric effect in order to track the displacement of a flexible system. For fundamentals of structural piezoelectricity, see, for example, Yang, Preumont, Safari and Akdogan, and Moheimani and Fleming,^1^, ^2^, ^3^, ^4^ particularly, displacement tracking, that is, the control of structural displacements, has gained an increasing interest in the last years in literature and engineering practice. Shape control denotes a particular case of displacement tracking, namely, that the desired displacement of the flexible system or at least the deflection at several locations shall be nullified. For literature overviews on shape control and displacement tracking, the reader is referred to Irschik, Irschik et al., Irschik and Krommer,^5^, ^6^, ^7^ and also Irschik et al.^8^ for recent results on displacement tracking of predeformed structures. Shape control and displacement tracking are feed‐forward control methods, where one asks for the control actuation in order that a certain desired displacement field is obtained. In case of piezoelectric control elements, one asks for the actuating electric field and its spatial distribution. It is assumed that the imposed load (given boundary excitations, imposed forces, applied temperature distributions, or imposed electric fields) as well as the parameters of the structure under consideration is completely known. For the piezoelectric control actuation, the converse piezoelectric effect, or an analogous physical actuation effect, is utilized. In theoretical considerations, the influence of the much smaller direct piezoelectric effect, which is frequently exploited in structural health monitoring for the sake of measuring structural displacements, is usually neglected, or it is taken into account in an approximate manner in the actuation context. The direct piezoelectric effect is needed for automatic control algorithms, which however are not in the focus of the present contribution but which should be superimposed when the above assumptions for successful feed‐forward control are not satisfied in a sufficient manner.

Driven by the recent encouraging technology development in the field of piezoelectric transducers and as a problem that is complementary to the above problem of controlling displacements, our group in the last years has studied the question, how to design piezoelectric control devices, so that a certain stress distribution can be achieved. Surprisingly, to our best knowledge, other groups hardly discussed this stress control problem in the open literature, although stress is known to be the main factor for failure and breakdown of structures, see Weber and Basu et al.^9^, ^10^ Exceeding a certain critical stress level will cause irreparable damage and structural failure already under static conditions. Under a periodic dynamic excitation, damage under a relatively small number of stress cycles will appear after this critical stress level has been approached, which is called low‐cycle fatigue. But, depending on the type of material under consideration, this critical stress level is known to decrease with the number of stress cycles. The corresponding structural failure under a high number of stress cycles at a lower stress level is known as high‐cycle fatigue. For fundamentals on fatigue, the reader is referred, for example, to Suresh, Schijve,^11^, ^12^ and Weisshaar.^13^

A first theoretical three‐dimensional (3D) framework for stress control has been formulated in Irschik,^14^ where stress control and displacement tracking strategies have been discussed at a continuum mechanics level, see also, for example, Irschik et al.^15^ for a short account on 3D dynamic stress compensation. More recently, Schoeftner and Irschik^16^ showed how to achieve a desired stress distribution for a base‐excited one‐dimensional bar with continuously distributed mass and stiffness by a proper temporal and spatial distribution of the piezoelectric actuation. Control of stress resultants, such as of bending moments, in piezoelectrically actuated continuous beam‐type structures has been treated in Schoeftner.^17^ In Irschik, Irschik et al., Schoeftner and Irschik, and Schoeftner,^14^, ^15^, ^16^, ^17^ first 3D and 1D theoretical and numerical results for the feed‐forward stress control by piezoelectric actuation have been presented, where it has been assumed that the necessary spatial and temporal distribution of the piezoelectric actuation can be realized everywhere in the structure under consideration. No experimental results have been presented so far to validate these theoretical findings in reality. The latter findings, which were obtained at a continuum mechanics level, however have given us confidence for designing laboratory experiments, which can be modeled at the level of structural mechanics and which can give clear evidence for the appropriateness of the concept of controlling stresses in structures by piezoelectric actuation. The first outcomes of these laboratory experiments are presented subsequently, where the corresponding structural system is described in detail below. In contrast to our previous theoretical considerations in Irschik, Irschik et al., Schoeftner and Irschik, and Schoeftner,^14^, ^15^, ^16^, ^17^ only a part of the considered system is subjected to the piezoelectric control actuation, and only the stress in that part is controlled. Hence, our subsequent formulations are novel also from a theoretical point of view.

**Figure 1:**
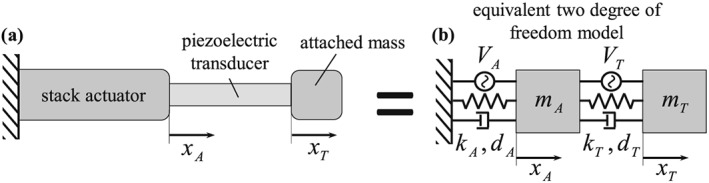
(a) Sketch of a piezoelectric transducer with attached mass, actuated by a piezoelectric stack actuator; (b) two‐degree‐of‐freedom model

In the present contribution, we intend to annihilate the longitudinal stress of a piezoelectric control device, which we call the piezoelectric transducer. Because the latter has section‐wise constant properties and only longitudinal vibrations are considered, the axial stress can be assumed to be uniformly distributed over the cross section and to be proportional to the internal force of each transducer. One end of the piezoelectric transducer is connected to a single point mass, whereas the other end is connected to a piezoelectric stack actuator, which serves as a robust excitation source of the transducer, see Figure 1 below.

Our paper is organized as follows. First, the system is approximated as a two‐degree‐of‐freedom (2DOF) system, where the two masses of the model replace the masses of the stack actuator, of the piezoelectric transducer and the end mass, and where the dynamic interaction between stack actuator and piezoelectric transducer is taken into account. Applying Newton's fundamental law of dynamics to the free‐body diagrams of the two equivalent masses, we set up their equations of motion not only in terms of the accelerations of the masses but also in terms of the internal forces in the stack actuator and in the piezoelectric transducer. The two equations of motion are accompanied by two linear constitutive equations, which relate the internal forces with the displacements and the velocities of the single masses and with the electric fields in the two elements. In the present structural mechanics framework, where we neglect the direct piezoelectric effect and we assume the axial component of the electric field to be predominant in the two piezoelectric devices, the electric fields are replaced by corresponding imposed electric voltages. Assuming that the motion of the system starts from rest and transforming the four relations into the Laplace domain, we eventually find a theoretical condition, how to electrically actuate the piezoelectric transducer in a feed‐forward framework, such that the internal force in the transducer, that is, the axial stress, is completely nullified. We first demonstrate in a numerical study that our theory yields the expected results, that is, that the force of the piezoelectric transducer vanishes in the numerical experiments when the theoretically derived piezoelectric actuation is applied to the transducer. As an important by‐product, our theoretical results demonstrate that it would not even be necessary to identify the numerical values of the parameters of the 2DOF model from the corresponding laboratory experiment, substituting the identified values afterwards into the theoretical solution, but that it is sufficient to perform preliminary transfer function measurements. This is because we succeed in identifying the theoretical stress control solution as the ratio of specific transfer functions of the system, which can be experimentally determined in the frequency domain easily.

Then, we realize the laboratory setup, for which we identify the parameters of the corresponding 2DOF model on the one hand side and we determine the required transfer functions at a low excitation level on the other hand side. The experimental setup eventually verifies our proposed stress control method: We show that the piezoelectric transducer, as long as the theoretically derived piezoelectric actuation signal for stress control is applied (control on), remains undamaged during a 30‐min test run. In contrast, in the uncontrolled configuration (control off), the internal force in the piezoelectric transducer exceeds a certain critical limit after a comparatively low number of cycles and thus is irreparably damaged. This failure results in a significant lowering of the frequency response peak of the system, and a crack in the piezoelectric transducer becomes clearly visible. The results of the tests are described in some detail. The paper ends with concluding remarks and a short outlook.

## 2 MODELING AND THEORETICAL FEED‐FORWARD STRESS CONTROL

In this section, we derive theoretical conditions for a successful feed‐forward (open loop) control of stress in a structural element. As already mentioned, we intend to annihilate the longitudinal stress in a comparatively light piezoelectric transducer (subscript *T*), which is actuated by a more massive piezoelectric stack actuator (subscript *A*). The stack is connected to the transducer, where at the free end of the piezoelectric transducer, there is a single attached mass, see Figure 1a.

The piezoelectric elements in Figure 1a are characterized by continuously distributed mass, stiffness, and damping. We now discretize the system in Figure 1a as a 2DOF system, see Figure 1b, where we follow the usual procedures and assumptions of structural mechanics. The two single masses of this model, which replace the stack actuator, the piezoelectric transducer, and the single attached mass, are denoted by *m*_*A*_ and *m*_*T*_, respectively. Note that the dynamic interaction between stack actuator and piezoelectric transducer is taken into account in the 2DOF model.

Moreover, because the two piezoelectric elements have section‐wise constant properties each and because only longitudinal vibrations are considered, the axial stresses are assumed to be uniformly distributed over the respective cross sections. Thus, the notion of stresses can be directly replaced by the internal forces in the transducer, *F*_*T*_, and in the stack actuator, *F*_*A*_, respectively. Hence, our goal is to nullify the internal force *F*_*T*_ by a control electric field that is applied to the piezoelectric transducer, in the presence of a given electric field in the stack actuator.

In order to find a theoretical solution, Newton's law of dynamics is applied to the two effective masses first. In the Laplace domain, these two equations of motion read as follows:

(1) $$m_{A}\;s^{2}\;x_{A}=-F_{A}+F_{T},$$

(2) $$m_{T}\;s^{2}\;x_{T}=-F_{T}.$$

**Figure 2:**
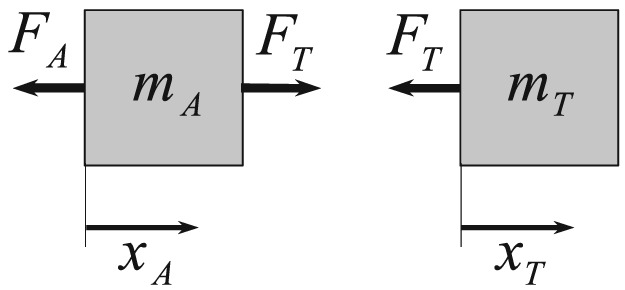
Free‐body diagram of the two‐degree‐of‐freedom model

The coordinate of the Laplace transform is denoted by *s*, and the Laplace‐transformed absolute displacements of the two effective masses are *x*_*T*_ and *x*_*A*_, respectively. For the free‐body diagrams leading to Equations (1) and (2), the reader is referred to Figure 2.

The two equations of motion (1) and (2) are accompanied by two linear constitutive equations, which, at the structural mechanics level, relate the internal forces with the displacements and the dominant electric field components. We consider linear viscoelastic behavior of the piezoelectric elements, in order to include light damping. In the present structural mechanics framework, neglecting the direct piezoelectric effect, and because the axial component of the electric field is predominant in the two piezoelectric devices, the electric fields are replaced by corresponding imposed electric voltages, see the sketch of the 2DOF system in Figure 1b. The constitutive behavior thus can be formulated as

(3) $$F_{T}=\left(k_{T}+s\;d_{T}\right)\left(x_{T}-x_{A}\right)+c_{T}V_{T},$$

(4) $$F_{A}=\left(k_{A}+s\;d_{A}\right)\;x_{A}+c_{A}V_{A}.$$

The elastic stiffness of an element is denoted by *k*, and *d* is a viscous material parameter. The Laplace‐transformed electric voltages applied to the two piezoelectric elements are denoted as *V*, and the effective piezoelectric constants at the structural mechanics level are denoted by *c*.

The four relations stated in Equations (1)–(4) can be used in various directions. A direct usage is to assume that the electric voltages in both piezoelectric elements be given and to compute the corresponding two displacements and two internal forces. Special direct solutions that are of interest for our later derivations follow by assuming that one of the two electric voltages in the two piezoelectric elements does vanish and to compute corresponding transfer functions. The notion of transfer function refers to the ratio between the Laplace transform of a certain output entity (superscripts *x*, *v*, and *F* for displacement, velocity, and force, respectively) and the Laplace‐transformed corresponding excitation. Particularly, let $G_{\mathrm{TA}}^{x}$ and $G_{\mathrm{TA}}^{v}$ be the transfer functions for the displacement and for the velocity of the end mass due to *V*_*A*_, when there is *V*_*T*_ = 0, and let $G_{\mathrm{TT}}^{x}$ and $G_{\mathrm{TT}}^{v}$ be the transfer function for the displacement due to *V*_*T*_, when *V*_*A*_ = 0,

(5) $$G_{\mathrm{TA}}^{x}=\frac{x_{T}}{V_{A}},\;G_{\mathrm{TT}}^{x}=\frac{x_{T}}{V_{T}},\;G_{\mathrm{TA}}^{v}=\frac{{\mathrm{sx}}_{T}}{V_{A}},\;G_{\mathrm{TT}}^{v}=\frac{{\mathrm{sx}}_{T}}{V_{T}}.$$

Then, it turns out that the ratio *χ*_*T*_ of the latter transfer functions becomes

(6) $$\chi_{T}=\frac{G_{\mathrm{TA}}^{x}}{G_{\mathrm{TT}}^{x}}=\frac{G_{\mathrm{TA}}^{v}}{G_{\mathrm{TT}}^{v}}=\frac{c_{A}\left(k_{T}+d_{T}s\right)}{c_{T}\left(m_{A}s^{2}+d_{A}s+k_{A}\right)}.$$

In a similar manner, we define the transfer function of the transducer force as the quotient of the Laplace‐transformed internal force *F*_*T*_ to the input voltages *V*_*A*_ (when *V*_*T*_ = 0) and *V*_*T*_ (when *V*_*A*_ = 0) as

(7) $$G_{\mathrm{TA}}^{F}=\frac{F_{T}}{V_{A}},\;G_{\mathrm{TT}}^{F}=\frac{F_{T}}{V_{T}}.$$

In Equation (6), the ratio is equal to the ratio of the corresponding transfer functions for the velocities of the mass and of the corresponding transfer functions for the internal forces in the piezoelectric transducer. The former fact holds, because, in the Laplace domain, velocity is obtained as *sx*_*T*_, and the latter follows from Equation (2). Note that the ratio *χ*_*T*_ can be obtained from laboratory experiments in the frequency domain under low‐level excitations in a straightforward manner. Another interesting case in the present context is the behavior of the system, when we set *m*_*T*_ = 0 in Equations (1)–(4). In reality, this corresponds to a setup, in which the piezoelectric transducer and the attached end mass are absent, which will hardly change the value of the effective mass of the model *m*_*A*_, because the stack actuator is taken as comparatively heavy. Anyway, setting *m*_*T*_ = 0 in Equation (2), from which follows *F*_*T*_ = 0, and inserting Equation (4) into Equation (1), the transfer function for the displacement of the mass *m*_*A*_ due to *V*_*A*_ becomes

(8) $${\left.G_{\mathrm{AA}}^{x}\right|}_{m_{T}=0}=-\frac{c_{A}}{m_{A}s^{2}+d_{A}s+k_{A}}.$$

The transfer function for the internal force in the stack actuator reads, see also Equations (1) and (8):

(9) $${\left.G_{\mathrm{AA}}^{F}\right|}_{m_{T}=0}=\frac{m_{A}s^{2}\;c_{A}}{m_{A}s^{2}+d_{A}s+k_{A}}.$$

Solutions of so‐called inverse problems related to Equations (1)–(4) also can be easily found, too. Shape control and displacement tracking are treated by requiring that the displacements should become certain desired functions of *s*, for example, that they should vanish; then, the electrical voltages *V*_*A*_, *V*_*T*_ and the internal forces *F*_*A*_, *F*_*T*_ are taken as the four unknowns. For our present stress control problem, we assume that the actuation voltage *V*_*A*_ in the stack actuator is given, and we treat the voltage *V*_*T*_ in the piezoelectric transducer as an unknown quantity, which must assure that our present control goal is satisfied, namely, that the internal force in the piezoelectric transducer should vanish,

(10) $$F_{T}=0.$$

Hence,

(11) $$x_{T}=0,$$

follows directly from Equation (2), and

(12) $$x_{A}=-\frac{c_{A}}{m_{A}s^{2}+d_{A}s+k_{A}}\;V_{A}={\left.G_{\mathrm{AA}}^{x}\right|}_{m_{T}=0}\;V_{A}$$

follows from Equations (1), (4), and (10). Inserting Equation (12) into Equation (2) yields the stack actuation force

(13) $$F_{A}=\frac{m_{A}s^{2}c_{A}}{m_{A}s^{2}+d_{A}s+k_{A}}\;V_{A}={\left.G_{\mathrm{AA}}^{F}\right|}_{m_{T}=0}\;V_{A},$$

and substitution of Equations (10)–(12) into Equation (3) yields

(14) $$V_{T}=-\chi_{T}\;V_{A}\;\text{with}\;\chi_{T}=\frac{c_{A}\left(k_{T}+s\;d_{T}\right)}{c_{T}\left(m_{A}s^{2}+k_{A}+s\;d_{A}\right)}.$$

We note that an alternative formulation and a solution for the transducer voltage in the time domain, which also takes into account the initial conditions, are presented in the Appendix. Here, we are only interested in the steady‐state solution, so initial disturbances will be attenuated after a while. It is interesting to note that the attached block *m*_*T*_ does not affect the outcome, see Equation (14) or Equation (34).

Furthermore, we have to remark here that the annihilation of stress does not necessarily mean also the annihilation of the displacement in general, as Equations (10) and (11) suggest. Here, we are interested in preventing the end mass to move; according to Newton's law, no force may act on the mass. Because we do not allow any external forces, a vanishing transducer force *F*_*T*_ = 0 means the end mass *m*_*T*_ to be at rest.

The relation in Equation (14) represents the feed‐forward solution of our stress control problem, that is, the Laplace transform of the necessary electric control voltage in order that the internal force in the piezoelectric transducer vanishes. An interesting approximate form of the control law in Equation (14), which is valid in the lower frequency domain, is obtained by neglecting the frequency dependent components:

(15) $$V_{T,\text{sta}}=-\chi_{T}\left(0\right)V_{A}\;\text{with}\;\chi_{T}\left(0\right)=\frac{k_{T}c_{A}}{k_{A}c_{T}}.$$

In the following, this is shortly denoted as static solution and used for comparison sake.

## 3 NUMERICAL RESULTS FOR STRESS CONTROL

### 3.1 Validation of the parameters

**Figure 3:**
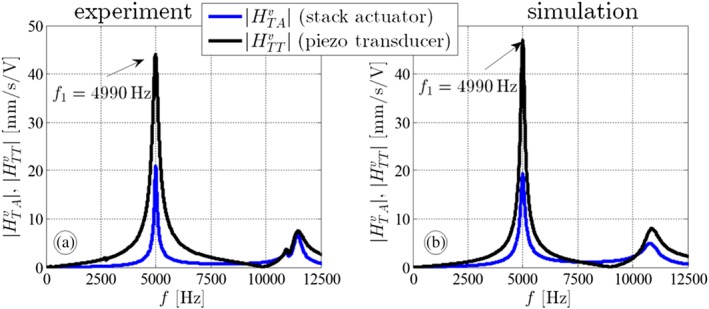
Transfer functions (black: stack actuation, blue: piezoelectric transducer actuation) of (a) the experiment and (b) the simulation model with the identified parameters listed in Table 1

In this section, we validate and parameterize our mathematical model. This can be done either by recalculation (e.g., the stiffness is computed by means of the geometry and the Young's modulus) or by relying on the manufacturers datasheet (for the piezoelectric transducer^18^ and for the stack actuator^19^). In our study, we weighed the masses of the stack, the transducer, and the attached mass and used the stiffness values and the piezoelectric coefficients from the manufacturer's datasheet in a first step. Then, in a later step, we compared the frequency response functions (FRFs) $H_{\mathrm{TA}}^{v}\left(\omega \right)={\left.G_{\mathrm{TA}}^{v}\right|}_{s=j\;\omega }$ and $H_{\mathrm{TT}}^{v}\left(\omega \right)={\left.G_{\mathrm{TT}}^{v}\right|}_{s=j\;\omega }$ of the simulation results to the experimental ones. If the results do not match properly, we modified the stiffness, mass, and damping values until we obtain a good agreement to the experimental FRFs. Hence, also for our low‐dimensional model, the limit of acceptability is considered to be 8000 Hz, which is between the first and second eigenfrequency. It is noted that the Fourier transform *H*(*ω*) can be considered as a special form of the Laplace transform *G*(*s*) taking the values on the positive imaginary axis only, see Shin and Hammond.^20^ We further modified the system parameters for the 2DOF model in order to find a better match between simulation and experimental results, see Figure 3. Two longitudinal vibration modes are visible: The fundamental eigenfrequency is at *f*_1_ = 4990 Hz and the second one at *f*_2_ ≈ 11500 Hz. For the first mode, the transducer and actuator mass vibrate in phase (when the motion/velocity of the actuator mass is much smaller, see the blue peak in Figure 3), in contrast to the antiphase oscillations for the second mode. The higher eigenmodes are irrelevant for our study because the third mode is hardly visible in the diagram and the dynamics beyond the second mode are out of our interest. The validated and modified parameters for the setup are listed in Table 1.

**Table 1:** Parameters used in the numerical study

Variable (unit)	Value
*m*_*A*_ (kg)	0.156
*m*_*T*_ (kg)	0.109
*k*_*A*_ (N/m)	495 ⋅ 10^6^
*k*_*T*_ (N/m)	156 ⋅ 10^6^
*d*_*A*_ (Ns/m)	259.84
*d*_*T*_ (Ns/m)	224.00
*c*_*A*_ (N/V)	8.12
*c*_*T*_ (N/V)	9

### 3.2 Three methods for stress control

**Figure 4:**
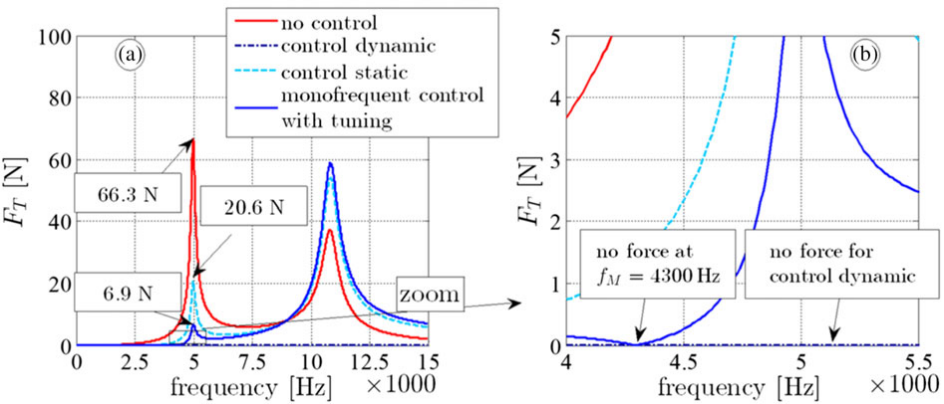
(a,b) Frequency response *F*_*T*_ due to the actuation amplitude *V*_*A*_ = 1 V, (c) necessary transducer amplitude *V*_*T*_ (red: without control *V*_*T*_ = 0 V, dark blue dashdot: dynamic control, see Equation (14), light blue dashed: static control, see Equation (15), blue: monofrequent control with tuning, see Equation (16))

In this section, we present three feed‐forward control algorithms. These are denoted as dynamic control, static control, and monofrequent control with tuning and explained in the following:

- Dynamic control: The transfer function Equation (14) is applied for the piezo control voltage *V*_*T*,*dyn*_(*s*) =  − *χ*_*T*_(*s*) *V*_*A*_(*s*), which should yield (theoretically) a perfect annihilation of the mass displacement and of the transducer force, see Equations (10) and (11). This should hold for any desired *s* = *jω*.
- Static control: The Laplace variable *s* is set to 0, so the transfer function in Equation (14) becomes a constant, see Equation (15), *V*_*T*,*sta*_(*s*) =  − *χ*_*T*_(0)*V*_*A*_(*s*). It turns out that this control voltage yields a very low displacement of the attached mass and of the transducer force in the low‐frequency domain, see Figure 4.
- Monofrequent control with tuning: Here, we are mainly interested in annihilating the transducer force only at *f* = 4300 Hz, from which follows:

(16) $$V_{T,\text{mono}}\left(s\right)=-\chi_{T}\left(j\omega_{M}\right)\;V_{A}\left(s\right).$$

We split up the complex‐valued function *χ*_*T*_(*jω*_*M*_) into *χ*_*T*_(*jω*_*M*_) = *χ*_*T*_(0)*F*(*jω*_*M*_), which clarifies that the static portion *χ*_*T*_(0) is real valued and the function *F*(*jω*_*M*_) is complex valued in general. Inserting Equations (6) and (15) into *F*(*jω*_*M*_) = *χ*_*T*_(*jω*_*M*_)/*χ*_*T*_(0) and setting *s* = *jω*_*M*_, one finds for *F*(*jω*_*M*_),

(17) $$\begin{array}{l} F\left(j\omega_{M}\right)=\frac{1+\left(d_{T}/k_{T}\right)j\omega_{M}}{\left[-\left(m_{A}/k_{A}\right)m_{A}\omega_{M}^{2}+\left(d_{A}/k_{A}\right)j\omega_{M}+1\right]},\\ \text{with}\;\omega_{M}=2\pi f_{M},\;f_{M}=4300\;\mathrm{Hz}. \end{array}$$

The alternative representation of the function $F\left(j\omega_{M}\right)=A^{*}e^{j\varphi^{*}}$ demonstrates that *F*(*jω*_*M*_) can be split up into the amplitude A^*^ that represents the matching gain, whereas the complex phase angle φ^*^ represents a phase shift in the frequency domain; that is, in the time domain, this means a time delay of the system. From Equations (15) and (17), it follows for *χ*_*T*_(*jω*_*M*_) in Equation (16),

(18) $$\chi_{T}\left(j\omega_{M}\right)=\frac{k_{T}c_{A}}{k_{A}c_{T}}\;\frac{1+\left(d_{T}/k_{T}\right)j\omega_{M}}{\left[-\left(m_{A}/k_{A}\right)m_{A}\omega_{M}^{2}+\left(d_{A}/k_{A}\right)j\omega_{M}+1\right]}.$$

One observes from Equation (18) that static control (see Equation (15)) is a special case of monofrequent control with *ω*_*M*_ = 0, that is, *F*(0) = 1.

### 3.3 Harmonic analysis for stress control

As a result of numerical computations, Figure 4 shows the amplitude of the transducer force *F*_*T*_ if the piezo transducers voltage is not present (i.e., *V*_*T*_ = 0V [red], control off) and for the three above control laws (control dynamic: dark blue dashdot, static control: light blue dashed, monofrequent control with tuning: blue). The actuation amplitude is *V*_*A*_ = 1 V. The corresponding transducer voltage signals *V*_*T*_ for dynamic, static, and monofrequent control are obtained by Equations (14)–(16), see Figure 4c. However, the dynamic control method is the only frequency‐dependent voltage and represents a transfer function with a peak at $f=\sqrt{k_{A}/m_{A}}/\left(2\pi \right)=8965\;\mathrm{Hz}$, see Equation (14). One recognizes the resonance frequencies at *f*_1_ = 4990 Hz and *f*_2_ ≈ 11000 Hz. The *dynamic control method*, Equation (14), yields a perfect annihilation of the transducer force (blue): The response is 0 for the whole frequency range. If the static control formula is applied for stress control, Equation (15), the force can be significantly reduced for frequencies *f* < 7500 Hz in comparison with the uncontrolled configuration. Especially, the force at the first eigenfrequency *f*_1_ is lowered by 69% (from 66.3 to 20.6 N/V). In the higher frequency domain, however, this control method fails: The response at the second eigenfrequency is even amplified. *Monofrequent control with tuning*, see Equation (16), shows that the transducer force vanishes at the matching frequency *f*_*M*_ = 4300 Hz. As long as the excitation frequency is close to the matching frequency, one observes a substantial force reduction: The force at the eigenfrequency is only 6.9 N/V. Assuming a discrepancy of the matching and the excitation frequency still leads to a sufficient reduction of the transducer force: Changing the excitation frequency to 4600 Hz (i.e., 7% higher than the matching frequency) yields *F*_*T*_ = 0.4 N, which is still much lower than the amplitude without control or if the *static control* method is applied.

Nevertheless, for the low‐frequency regime, the method *static control* works well. An improvement can be achieved by means of *monofrequent control with tuning*, where the control tracking variable *F*_*T*_ can be annihilated at the specific frequency *ω*_*M*_.

## 4 EXPERIMENTAL RESULTS FOR STRESS CONTROL

### 4.1 Description of the experimental setup

**Figure 5:**
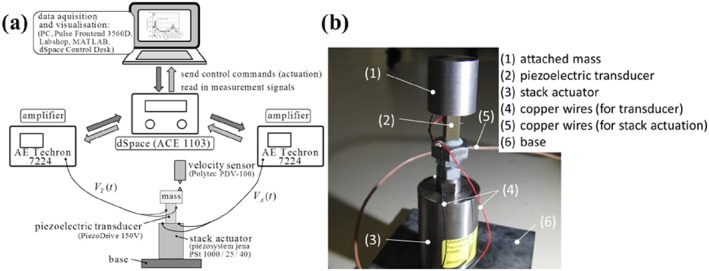
(a) Schematic presentation; (b) photograph and electronic devices for the experimental setup

A photograph and a scheme of the experimental setup are shown in Figure 5. Figure 5b shows the attached mass (1), which is glued onto the piezoelectric transducer (2). The transducers are an SA Series 150‐V Piezo Stack Actuator SA070718 from the company piezo drive.^19^ The piezoelectric stack actuator (3) (Piezocomposite—stack‐type actuator series PStVS with preload, PSt 1000/25/40 VS 35, piezosystem jena^18^) serves as the excitation source and is rigidly connected to the lower surface of the transducer. The copper wires transmit the excitation *V*_*A*_ and the control signal *V*_*T*_ to the stack actuator and to the transducer, respectively. As glue material, an electrically isolating two‐component adhesive (Loctite Hysol 9466 A&B) is used. The electrodes of the transducer and of the stack actuator are linked to the linear power amplifiers (AE Techron 7224 power amplifier), which amplify the input signal from the control unit by a factor 20. Lossless copper wires transmit the necessary voltage signals *V*_*A*_ and *V*_*T*_ to the stack actuator and to the transducer electrodes of the actuation.

Figure 5 shows the vertical arrangement of our setup. The consideration of gravity effects in Equations (1) and (2) does not play a significant role in our study, because the influence of gravity is much smaller than the deflection due to stack actuation, in particular close to the first resonance. In order to verify our stress control theory experimentally, the velocity of the attached mass *v*_*T*_ is measured (device Polytec PDV‐100). We recalculate the axial transducer force, where the attached mass is glued onto the transducer cross section, as *F*_*T*_ = *m*_*T*_*s*^2^*x*_*T*_, see Equation (2). The excitation and control signals in time‐domain *V*_*A*_, *V*_*T*_ are generated by utilizing dSpace ACE 1103 (hardware) and dSpace Control Desk (software). The corresponding real‐time C‐code (i.e., Equations (14)–(16) with the corresponding analog–digital output blocks for the communication is generated from a MATLAB/Simulink model, in which the feed‐forward control model is implemented. The sampling time of the dSpace hardware is *T*_s_ = 1/20,000 s; this means that the sampling frequency is *f*_s_ = 20,000 Hz. PULSE Front‐end 3560D from Brüel&Kjær is used as a measurement device to read in the actuation and the sensor signals. The measurement software is PULSE LabShop (version 18). The sampling time of the measurement device is *T*_s_ = 1/64,000 s.

### 4.2 Description of the test procedure

**Figure 6:**
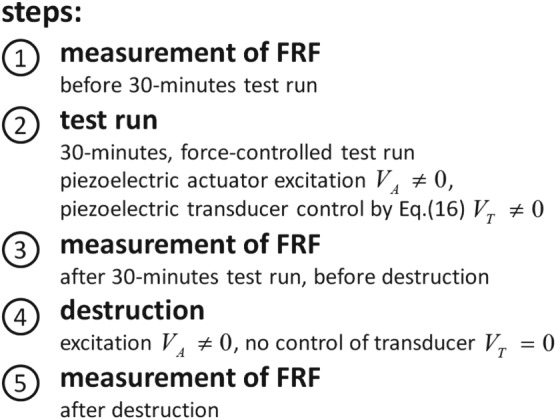
The five steps of the test procedure

In this section, we experimentally verify the force control theory developed in Section 2. We show that a force‐controlled transducer does not suffer any damage, whereas an uncontrolled setup suffers an irreparable mechanical breakdown. As an objective criterion, it is evident that a significant mechanical damage is related to a notable loss of stiffness; therefore, a significant change of the eigenfrequency is an indicator for the damage. The test procedure, which is divided into five steps, is depicted in Figure 6 and explained in the following.

#### 4.2.1 Step 1 ‐ FRF measurements before 30‐min test run

In a first step (Step 1), we measure the FRFs $H_{\mathrm{TA}}^{v}\left(\omega \right)$ and $H_{\mathrm{TT}}^{v}\left(\omega \right)$ of the undamaged 2DOF system (i.e., $H_{\mathrm{TA}}^{v}\left(\omega \right)$ is the amplitude of velocity of the attached mass, if the stack actuation is excited by *V*_*A*_ (with a short‐circuited piezo transducer *V*_*T*_ = 0), and $H_{\mathrm{TT}}^{v}\left(\omega \right)$ is the attached mass velocity if the piezoelectric transducer is harmonically excited by *V*_*T*_ (with a short‐circuited stack actuator *V*_*A*_ = 0)).

#### 4.2.2 Step 2 ‐ 30‐min force‐controlled test run

Then, we perform a 30‐min test run by controlling the force of the piezoelectric transducer using *monofrequent control with tuning*, where we used the measured transfer functions $H_{\mathrm{TA}}^{v}\left(\omega \right)$ and $H_{\mathrm{TT}}^{v}\left(\omega \right)$ from Section 4.2.1 for calculating the desired transfer ratio *χ*_*T*_ at *ω* = *ω*_*M*_, compare Equations (6) and (14). Among the three control methods that we introduced in Section 3.2, we prefer monofrequent control because it represents an acceptable compromise between satisfying stress control results on the one hand and it is relatively insensitive to parameter uncertainties on the other hand. The excitation (stack actuation) is *V*_*A*_ = 40 sin(*ω*_*M*_*t*), with an excitation frequency *f*_*M*_ = 4300 Hz that is close to the first resonance frequency. The control signal of the piezoelectric transducer theoretically is realized by transforming Equation (16) back into time domain. During the half‐hour test run (Step 2), the experimental setup is subjected to 7.74 ⋅ 10^6^ stress cycles (i.e., 30 min at 4300 Hz) when the fatigue stress limit (=endurance limit) is achieved for ceramic materials.

#### 4.2.3 Step 3 ‐ FRF measurements after 30‐min test run but before damage

After the test run, we have to prove that the transducer is not damaged. In addition to obvious cracks of the surface, we need to define an objective criterion. Hence, we measure the FRFs again.

#### 4.2.4 Step 4 ‐ Destruction phase

Step 4 is the destruction phase, when the same excitation voltage for the stack as in Phase 3 is applied. This time, the electrodes of the piezoelectric transducer are short‐circuited *V*_*T*_ = 0 V (control off). According to the manufacturer of the transducers,^18^ damage should occur if the transducer force exceeds 10–20% of the blocking force *F*_*block*_ ≈ 1,800 N.

#### 4.2.5 Step 5 ‐ FRF after destruction

In order to detect damage and failure, we perform an FRF analysis, observing changes of the system properties. Significant changes of the velocity response, in particular a reduction of the first resonance frequency and peak modifications, are clear indicators for the transducer damage.

### 4.3 Experimental results

**Figure 7:**
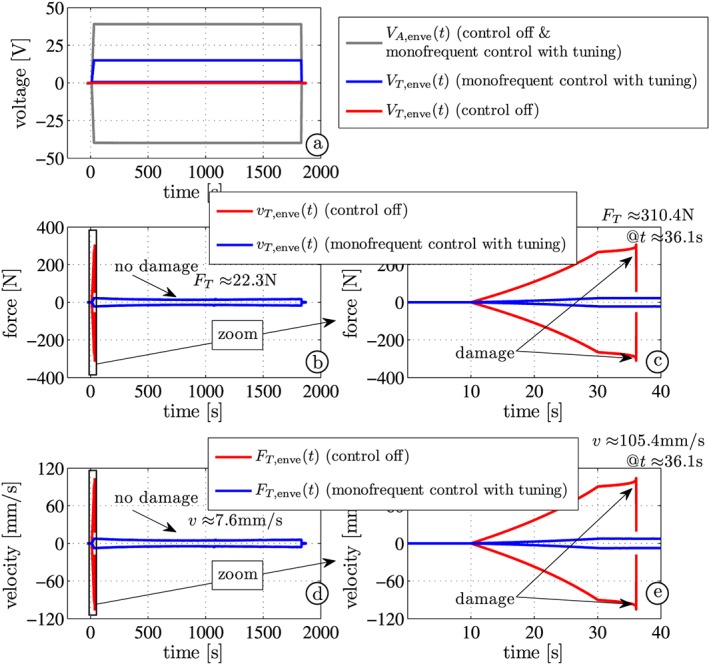
Envelope curves for (a) the voltage actuation and the piezoelectric control signal, (b) velocity of the attached mass, and (c) zoom (without control red, with control blue)

Figure 7 shows the experimental results of Steps 2 (30‐min test run in the controlled case *V*_*T*_ ≠ 0) and 4 (destruction phase for *V*_*T*_ = 0). The envelopes of the applied sinusoidal stack and piezo‐transducers voltages *V*_*A*,enve_ and *V*_*T*,enve_ are shown in Figure 7a (gray and red signals for the uncontrolled case, gray and blue signals for the case *monofrequent control with tuning*). Figure 7b–e shows the envelopes of the velocity and of the transducer force of the uncontrolled (Figure 7c and 7e) and the controlled (Figure 7b and 7d) configurations. One observes that the maximum velocity amplitude of the force‐controlled configuration is only *v*_max_ = 7.6 mm/s (Figure 7b, blue), which means a transducer force of *F*_*T* max_ = *m*_*T*_(2*π*4300)⋅0.0076 ≈ 22.3 N that is much lower than the necessary damage force *F*_damage_ ≈ 0.1 *F*_block_ = 180 N. It is noted that according to the manufacturer's datasheet, the limit tensile force is about 10% to 20% of the blocking force.^18^ If the control method is deactivated from the beginning, one observes a very large velocity amplitude peak *v* = 105.6 mm/s, which means a transducers force of *F*_*T*_ ≈ 310.4 N (17% of the blocking force, see Figure 7c, red). Indeed, at *t* = 36.1 s, the transducer suffers damage, see Figure 7.

**Figure 8:**
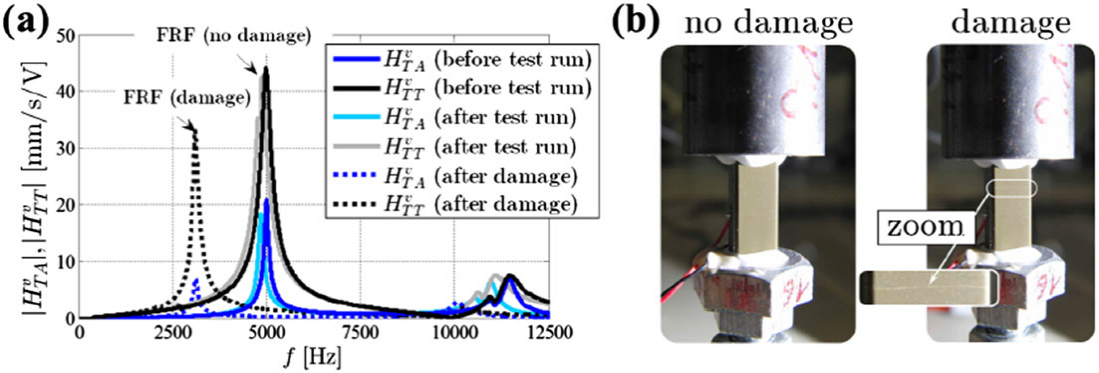
(a) Frequency response functions before (Step 1: blue/black), after the 30‐min test run (Step 3: light blue/gray), and after the damage (Step 5: dashed blue/black). (b) The test specimen shows the horizontal crack of the piezoelectric transducer (compare the damaged transducer (right) to the undamaged transducer (left))

Finally, the FRFs for Steps 1, 3, and 5 are shown in Figure 8a. For Steps 1 and 3, the change of the first eigenfrequency before and immediately after the 30‐min test run is negligible; that is, there is no proof for damage. In Step 5, however, one recognizes a remarkable shift of the first eigenfrequency from 4990 Hz (undamaged configuration) to 3100 Hz (damaged configuration, dashed lines), which clearly indicates damage of the transducer. Further damage indicators are shown in Figure 8b (left: undamaged; right: damaged), where one can observe a horizontal crack line on the surface of the transducer.

## 5 CONCLUSION

A main goal of the present contribution is the experimental verification of feed‐forward stress control. We investigate, how a force‐excited piezoelectric transducer with an attached mass can be piezoelectrically actuated in such a manner, that the transducer force is nullified or at least considerably reduced despite a piezoelectric actuation in a stack actuator is present, see Figures 1 and 5. In a first step, we discretize this system as a 2DOF model. One degree of freedom stands for the stack actuator; the other degree of freedom models the attached mass and the piezoelectric transducer. Using this simple structural model, we first present a theoretical relation for the piezoelectric voltage actuation that is needed, such that the force in the transducer can be eliminated, see Equation (14). Interestingly enough, it turns out that the attached mass remains at rest in the controlled configuration, see Equation (11), which should give raise to various practically appealing applications. We then prove our theory by numerical computations. In these computations, it is demonstrated that it should be indeed possible in practice to nullify the transducer force for a given stack actuation, see Figure 4. Finally, an experimental setup proves our developed stress control method, see Figure 5. Our test specimen is harmonically excited close to the first resonance frequency. It is shown that the endurance stress limit (fatigue limit) is not exceeded and the device does not experience any damage, if the developed stress control method is applied. Contrary, the uncontrolled configuration suffers an irreparable mechanical damage, if the specimen exceeds a certain stress limit, see Figures 7 and 8. To the best knowledge of the authors, our study is the first experimental verification of feed‐forward stress control by piezoelectric actuation in the open literature. We understand it as a further step toward the development of so‐called ageless structures.

## APPENDIX 1

Finally we present an alternative derivation for our force control theory. Contrary to section 1 when all signals are considered as Laplace‐transformed signals, here the displacements *x*_*A*_, *x*_*T*_, the voltages *V*_*A*_, *V*_*T*_ and the forces *F*_*A*_, *F*_*T*_ (see Equations (19)–(34)) are time‐domain signals. We present a general solution for the necessary voltage actuation of the transducer, if the system does not start from rest and if the desired transducer force is non‐zero, see Equation (28).

Applying Newton's law for the two masses (see Figure 1) and solving for the acceleration, one finds

(19) $$x_{A,\mathrm{tt}}=\left(-F_{A}+F_{T}\right)/m_{A}$$

(20) $$x_{T,\mathrm{tt}}=-F_{T}/m_{T}.$$

The spring‐law (without damping) reads, see Figure 2

(21) $$F_{T}=k_{T}\left(x_{T}-x_{A}\right)+c_{T}V_{T}$$

(22) $$F_{A}=k_{A}\;x_{A}+c_{A}V_{A}$$

Multiplying Equation (19) by *k*_*A*_ and adding *c*_*A*_*V*_*A*,*tt*_ yields

(23) $$k_{A}x_{A,\mathrm{tt}}+c_{A}V_{A,\mathrm{tt}}=\left(-F_{A}+F_{T}\right)\frac{k_{A}}{m_{A}}+c_{A}V_{A,\mathrm{tt}},$$

and multiplying Equation (20) by *k*_*T*_ and adding (*c*_*T*_*V*_*T*,*tt*_ − *k*_*T*_*x*_*A*,*tt*_) yields

(24) $$k_{T}\left(x_{T,\mathrm{tt}}-x_{A,\mathrm{tt}}\right)+c_{T}V_{T,\mathrm{tt}}=-F_{T}\frac{k_{T}}{m_{T}}-k_{T}x_{A,\mathrm{tt}}+c_{T}V_{T,\mathrm{tt}}.$$

Note that the left hand sides of Equations (23) and (24) are *F*_*A*,*tt*_ and *F*_*T*,*tt*_, respectively. Substituting Equation (19) into (24) and replacing the left‐hand sides of Equations (23) and (24), one finds the following two coupled second order differential equations for the transducer force *F*_*T*_ and for the stack actuator force *F*_*A*_

(25) $$F_{A,\mathrm{tt}}+\frac{k_{A}}{m_{A}}F_{A}=F_{T}\frac{k_{A}}{m_{A}}+c_{A}V_{A,\mathrm{tt}}$$

(26) $$F_{T,\mathrm{tt}}+k_{T}\left(m_{T}^{-1}+m_{A}^{-1}\right)F_{T}=\frac{k_{T}}{m_{A}}F_{A}+c_{T}V_{T,\mathrm{tt}}.$$

Transforming Equations (25) and (26) into a fourth order differential equation for the piezoelectric transducer force yields *F*_*T*_(*t*), one finds

(27) $$c_{T}\frac{m_{A}}{k_{T}}V_{T,\text{tttt}}+c_{T}\frac{k_{A}}{k_{T}}V_{T,\mathrm{tt}}=-c_{A}V_{A,\mathrm{tt}}+\frac{m_{A}}{k_{T}}F_{T,\text{tttt}}+\left(\frac{k_{A}}{k_{T}}+\frac{m_{A}}{m_{T}}+1\right)F_{T,\mathrm{tt}}+\frac{k_{A}}{m_{T}}F_{T}.$$

Correction added on 18 March 2019, after first online publication: equations (27) and (28) have been corrected.

Equation (27) states how to choose the actuation voltage, if the transducer force *F*_*T*_(*t*) follows a certain path (in general *F*_*T*_(*t*) ≠ 0) and if the voltage actuation *V*_*A*_ (that is considered as disturbance source here) is known a‐priori. Performing integration with respect to time twice, we find the second order ordinary differential equation for the piezoelectric transducer voltage *V*_*T*_(*t*)

(28) $$c_{T}\frac{m_{A}}{k_{T}}V_{T,\text{tt}}+c_{T}\frac{k_{A}}{k_{T}}V_{T}=-c_{A}V_{A}+\frac{m_{A}}{k_{T}}F_{T,\mathrm{tt}}+\left(\frac{k_{A}}{k_{T}}+\frac{m_{A}}{m_{T}}+1\right)F_{T}+\frac{k_{A}}{m_{T}}\iint F_{T}\;dt\;dt+C_{1}t+C_{0},$$

[Link]which requires 2 initial conditions *V*_*T*_(0), *V*_*T*,*t*_(0) and the specification of the integration constants *C*_0_ and *C*_1_. Taking into account the spring law at *t* = 0

(29) $$\begin{array}{l} F_{T}\left(0\right)=k_{T}(x_{T}\left(0\right)-x_{A}\left(0\right))+c_{T}V_{T}\left(0\right)\\ \to V_{T}\left(0\right)=\frac{1}{c_{T}}[F_{T}\left(0\right)-k_{T}\left(x_{T}\left(0\right)-x_{A}\left(0\right)\right)] \end{array}$$

(30) $$\begin{array}{l} F_{T,t}\left(0\right)=k_{T}\left(x_{T,t}\left(0\right)-x_{A,t}\left(0\right)\right)+c_{T}V_{T,t}\left(0\right)\\ \to V_{T,t}\left(0\right)=\frac{1}{c_{T}}\left[F_{T,t}\left(0\right)-k_{T}\left(x_{T,t}\left(0\right)-x_{A,t}\left(0\right)\right)\right] \end{array}$$

one finds the initial conditions for *V*_*T*_(0), *V*_*T*,*t*_(0). The integration constants *C*_0_ and *C*_1_ can be calculated by further time‐derivatives of Equations (29) and (30), when *V*_*T*,*tt*_(0) and *V*_*T*,*ttt*_(0) from Equations (31) and (32)

(31) $$\begin{array}{l} F_{T,\mathrm{tt}}\left(0\right)=k_{T}\left(x_{T,\mathrm{tt}}\left(0\right)-x_{A,\mathrm{tt}}\left(0\right)\right)+c_{T}V_{T,\mathrm{tt}}\left(0\right)\\ \to V_{T,\mathrm{tt}}\left(0\right)=\frac{1}{c_{T}}\left[F_{T,\mathrm{tt}}\left(0\right)+\frac{k_{T}}{m_{T}}F_{T}\left(0\right)+\frac{k_{T}}{m_{A}}(k_{A}x_{A}\left(0\right)+c_{A}V_{A}\left(0\right))\right] \end{array}$$

and

(32) $$\begin{array}{l} F_{T,\mathrm{ttt}}\left(0\right)=k_{T}\left(x_{T,\mathrm{ttt}}\left(0\right)-x_{A,\mathrm{ttt}}\left(0\right)\right)+c_{T}V_{T,\mathrm{ttt}}\left(0\right)\\ \to V_{T,\mathrm{ttt}}\left(0\right)=\frac{1}{c_{T}}\left[F_{T,\mathrm{ttt}}\left(0\right)+\frac{k_{T}}{m_{T}}F_{T,t}\left(0\right)+\frac{k_{T}}{m_{A}}\left(k_{A}x_{A,t}\left(0\right)+c_{A}V_{A,t}\left(0\right)\right)\right] \end{array}$$

are inserted into Equation (28) and its time‐derivation.

A special case solution is found if all initial conditions are zero and one demands *F*_*T*_(*t*) to vanish (i.e. force annihilation). Then one finds from Equation (28)

(33) $$c_{T}\frac{m_{A}}{k_{T}}V_{T,\mathrm{tt}}+c_{T}\frac{k_{A}}{k_{T}}V_{T}=-c_{A}V_{A}\;\text{with}\;V_{T}\left(0\right)=V_{T,t}\left(0\right)=0$$

Transformation into the Laplace domain yields

(34) $${\hat{V}}_{T}=-\frac{c_{A}k_{T}}{c_{T}\left(m_{A}s^{2}+k_{A}\right)}{\hat{V}}_{A}$$

where the tilde‐symbol denotes the corresponding signals in the Laplace domain. One observes that Equation (34) is equal to Equation (14) if damping is neglected. Furthermore we see that the attached transducer mass *m*_*T*_ does not influence the solution. If the configuration does not start from rest, e.g. *x*_*T*_(0) ≠ 0, it can be shown by steps (28) to (34) that the steady‐state solution is not affected as long as a little amount of damping is considered which is always the case in reality (as in our experimental setup).
